# The Polarized Redistribution of the Contractile Vacuole to the Rear of the Cell is Critical for Streaming and is Regulated by PI(4,5)P2-Mediated Exocytosis

**DOI:** 10.3389/fcell.2021.765316

**Published:** 2022-07-19

**Authors:** Sana A. Fadil, Chris Janetopoulos

**Affiliations:** ^1^ Department of Biological Sciences, University of the Sciences in Philadelphia, Philadelphia, PA, United States; ^2^ Department of Natural product, Faculty of Pharmacy, King Abdulaziz University, Saudia Arabia; ^3^ The Science Research Institute, Albright College, Reading, PA, United States; ^4^ The Department of Cell Biology at Johns Hopkins University School of Medicine, Baltimore, MD, United States

**Keywords:** contractile vacuole, polarity, phosphoinositide (4, 5)-bisphosphate, signal relay, chemotaxis

## Abstract

*Dictyostelium discoideum* amoebae align in a head to tail manner during the process of streaming during fruiting body formation. The chemoattractant cAMP is the chemoattractant regulating cell migration during this process and is released from the rear of cells. The process by which this cAMP release occurs has eluded investigators for many decades, but new findings suggest that this release can occur through expulsion during contractile vacuole (CV) ejection. The CV is an organelle that performs several functions inside the cell including the regulation of osmolarity, and discharges its content via exocytosis. The CV localizes to the rear of the cell and appears to be part of the polarity network, with the localization under the influence of the plasma membrane (PM) lipids, including the phosphoinositides (PIs), among those is PI(4,5)P2, the most abundant PI on the PM. Research on *D. discoideum* and neutrophils have shown that PI(4,5)P2 is enriched at the rear of migrating cells. In several systems, it has been shown that the essential regulator of exocytosis is through the exocyst complex, mediated in part by PI(4,5)P2-binding. This review features the role of the CV complex in *D. discoideum* signaling with a focus on the role of PI(4,5)P2 in regulating CV exocytosis and localization. Many of the regulators of these processes are conserved during evolution, so the mechanisms controlling exocytosis and membrane trafficking in *D. discoideum* and mammalian cells will be discussed, highlighting their important functions in membrane trafficking and signaling in health and disease.

## Introduction

Chemotaxis is a form of migration where cells migrate directionally, typically towards a gradient of chemicals known as chemoattractants. During this process, cells often have asymmetric responses and develop a polarized morphology, where they have a distinct front and rear (Devreotes and Janetopoulos, 2003). In the model organism *Dictyostelium discoideum,* a soil-living amoeba, cells move towards one another in a head-to-tail fashion and align in streams (Kriebel et al., 2008). The cyclic adenosine monophosphate (cAMP) is the chemoattractant that leads to aggregation of the amoebas into multicellular structures during starvation conditions (Konijn et al., 1969; Goldbeter, 1975; Goldbeter, 2006). Although many researchers have studied intracellular signaling in *D. discoideum*, the mechanism by which cAMP release occurs, setting up a gradient at the rear of individual cells, has remained elusive. How does signal relay produce an effective, localized chemotactic response from the rear of a cell? The enzyme that synthesizes cAMP, adenylyl cyclase (ACA), has been reported to be in a gradient along the periphery of the cell, with more accumulated in the rear (Kriebel et al., 2008). ACA also appears to be enriched in intracellular vesicles within the cell and multivesicular bodies left behind migrating cells. In this same study, Kriebel et al. suggested that cAMP is released from the rear of migrating cells *via* these extracellular vesicles (Kriebel et al., 2018).

The main regulator of ACA, known as the Cytosolic Regulator of Adenylyl Cyclase (CRAC), contains a pleckstrin homology (PH) domain which regulates the translocation to the leading edge of the cell during each transient activation of ACA (Insall et al 1994; Lilly and Devreotes 1994; Parent et al., 1998; Ruchira et al., 2004). This PH domain of CRAC binds to PI(3,4)P2 and PI(3,4,5)P3, with these two PIs being synthesized at the very front of the cell. This PH domain is required for ACA function, suggesting that the membrane localization of CRAC brings the regulator in close proximity to ACA for activation (Ruchira et al., 2004). It is unclear how or if CRAC at the leading edge of the cell activates the ACA at the trailing edge. If it somehow leads to ACA activity at the rear, the cAMP will still need to be somehow released from the cell.

Interestingly, another potential regulator of cAMP signaling from the rear of the cell has emerged. Fadil et al. (2022) discovered that the CV is redistributed to the rear of migrating cells and regulates cell streaming and cAMP secretion. In this mechanism, CRAC recruitment and ACA activity at the leading edge can synthesize cAMP, which then would diffuse through the cytosol and be pumped into the CV network. The mechanism controlling the CV redistribution is still under investigation, however evidence is emerging that higher levels of PI(4,5)P2 and potentially other charged lipids may play a role in the CV localization. The CV appears to be part of the overall polarity network, localizing to areas of actomyosin contraction.

In addition to being targeted to particular regions of the periphery of the cell, once there, the CV regulates the discharge of water by a kiss-and-run exocytic event (Essid et al., 2012). Exocytosis is an essential membrane trafficking process that can discharge soluble and insoluble intracellular protein contents including neurotransmitters, hormones, and cytokines, as well as many other small molecules and metabolites into the extracellular space (Voets, 2000; Rettig and Neher, 2002; Sudhof, 2013; Imig et al., 2014). As described here, PI(4,5)P2, plays a role in many of the PM-related cellular activities, including regulated vesicle exocytosis (Wenk et al., 2001). PI(4,5)P2 has been found to be critical for exocytosis in several mammalian cell types and is involved in neuronal function and disrupted in several human diseases (Stokes et al., 1978; Cremona et al., 1999; Nandez et al., 2014; Li et al., 2020). This review describes the importance of the polarity network regulating CV localization and highlights the important role PI(4,5)P2 plays in mediating vesicle fusion and CV-PM interactions.

## Contractile Vacuoles and Exocytosis in *Dictyostelium discoideum*

Freshwater protists like the amoeba, Heliozoans, and many Ciliates regulate water’s penetration by a complex organelle responsible for their osmoregulation. This organelle is known as the contractile vacuole (Schneider, 1960; De Chastellier et al., 1978; Patterson, 1980; Hausmann and Patterson, 1984; Allen, 2000; Frankel, 2000). In *D. discoideum,* the CV organelle is composed of an extensive network of tubules and bladders linked to the PM. This is critical when the cell encounters hypotonic environments where maintaining osmoregulation is critical (Gerisch et al., 2002). The excess water accumulates in the tubules, filling the vacuoles, which then fuse with the PM to expel the water into the extracellular medium (Heuser et al., 1993). After ejection of water through the vacuole, the tubules elongate again and collect water for resuming the cycle. Late in the cycle, the CV ejects its content in a focal kiss-and-run exocytic event where the CV and PM transiently interconnect (Allen, 2000; Allen and Naitoh, 2002; Frankel, 2000; Essid et al., 2012; McKanna, 1973, 1976).

There are several critical proteins that control CV function, including calmodulin (Moniakis et al., 1995). In fact, antibodies against calmodulin were one of the first markers used to identify the CV in fixed cells (Gerisch et al., 2002; Zhu and Clarke, 1992). LvsA (large volume sphere), a protein that binds to calmodulin, has been shown to localize to the CV and is required for osmoregulation (Gerald et al., 2001; Malchow et al., 2006). Mutant cells missing the LvsA protein are osmo-sensitive and impaired in the vacuole discharge (Gerald et al., 2001). Another protein, Disgorgin, which is a GAP for Rab8a is also required for CV discharge mediated by fusion with the PM (Du et al., 2008). Disgorgin and LvsA, together with GTP hydrolysis by Rab8a, are also essential for the CV detachment from the PM after discharging its contents (Essid et al., 2012) while Rab2 and RabS have also been shown to be localize to the CV and be important for osmoregulation (Maringer et al., 2016). The distribution of adaptor proteins AP 1 (Lefkir et al., 2003) or AP 180 (Stavrou and O’Halloran, 2006) each caused disruption in osmoregulation. Proteins that govern this specialized organelle’s activities in *D. discoideum* have been conserved throughout evolution and many of their orthologs have been shown to regulate mammalian membrane trafficking (Duhon and Cardelli, 2002; Neuhaus et al., 2002).

The connections between the CV and the PM are regulated by the exocyst complex, and contain Rab GTPases localized to the CV, which assist in regulating fusion with the PM (Yeaman et al., 2001; Essid et al., 2012; He and Guo, 2009; Maringer et al., 2016). Exocytosis and vesicular transport are known to be regulated by PIs (Paolo et al., 2004). In particular, PI(4,5)P2 is involved in the priming of the vesicle to the targeted membrane and fusion step (Cremona et al., 1999; Paolo et al., 2004; Holz et al., 2000; James et al., 2008; Milosevic et al., 2005; Martin, 2001; Wenk et al., 2001). The exocyst complex is an octameric complex of the subunits Sec3, Sec5, Sec6, Sec8, Sec10, Sec15, Exo70, and Exo84 and contains other components, including the SNARE (soluble N-ethylmaleimide-sensitive factor (NSF) attachment protein receptors)- associated protein SecA that have also been shown to be required for the CV discharge function (Sriskanthadevan et al., 2009; Zanchi et al., 2010; Essid et al., 2012). Among different Rab GTPase proteins, RabD (Knetsch et al., 2001; Harris and Cardelli, 2002), Rab4 (Bush et al., 1994, 1996), Rab8a (Essid et al., 2012), and Rab11 (Harris et al., 2002) are identified as regulators for discharge function.

### The Role of Rear Contractile Vacuole in cAMP Secretion

**FIGURE 1:**
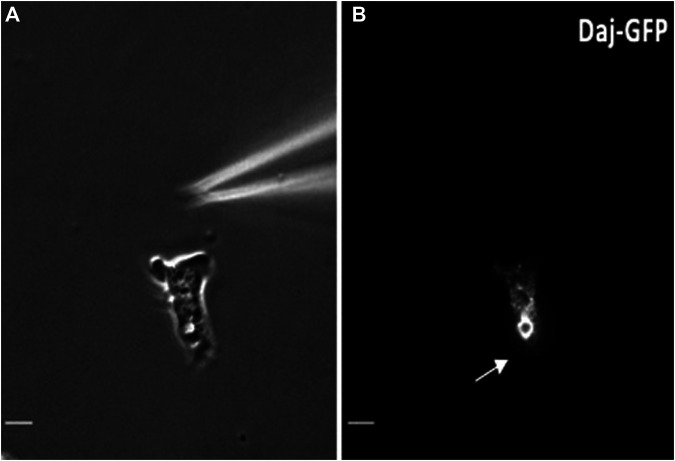
The CV is localized at the rear of migrating cells. **(A)** Phase contrast image of the CV at the rear of the polarized migrating cell. **(B)** The same cell as in 1C expressing Dajumin-GFP moving toward a micropipette filled with chemoattractant cAMP. Arrow indicates the localization of the CV. Courtesy of Fadil et al., 2022.

During the process of polarization, the CV redistributes to the rear of *D. discoideum* cells and is critical for the streaming phenomena mediated by the chemoattractant cAMP (Figure 1) (Fadil et al., 2022). The cAMP binds to the serpentine cAMP receptors, triggering heterotrimeric G signaling with the cell. Downstream responses include the activation of PI3 Kinase, which leads to the recruitment of the CRAC protein, triggering the synthesis of cAMP from ATP by ACA. In this new model, cAMP diffuses within the cytosol and is pumped into the CV network by the AbcC8 transporter (Fadil et al., 2022). The cell then discharges cAMP, likely along with Ca^+2^ (Fadil et al., 2022; Parkinson et al., 2014) from the cell’s rear through the CV vacuole tethered to PM. This provides the localized cAMP release from the back of the cells and supports the head to tail streaming characterized during early aggregation. The Dajumin-GFP (Gerisch et al., 2002) labeled the CV vacuoles and tubules and was localized to the rear of migrating cells (Figure 1). The cAMP transporter AbcC8 has recently been identified as the main cAMP transporter (Kriebel et al., 2018), and when fluorescently tagged, its localization mirrored that of Dajumin, localizing throughout the CV and tubules (Fadil et al., 2022). Thus, the presence of the AbcC8 transporter within the CV network provides a mechanism for cAMP to enter the CV tubules, with cAMP being released from the rear of the cell during the ejection phase of the CV cycle.

To confirm the importance of a functional CV in cell streaming, two different mutant cell lines were tested that have major defects in assembling a functional CV, Huntingtin null and LvsA null cells. The exact function of both proteins is not known, with evidence suggesting they are involved in membrane trafficking. In mammals, the huntingtin protein is critical for neuronal function, but like its ortholog, the role of this protein is not clear. While still having the ability to chemotax, both *D. discoideum* cell lines displayed decreased stability of head-to-tail cell contacts and lacked the normal streaming behavior seen in wild-type cells. Additionally, the periodic cAMP waves seen during early aggregation were disrupted, as was visualized using the cytosolic cAMP indicator, Flamindo2 (Fadil et al., 2022; Hashimura et al., 2019). Thus, the ability of the cells to perform signal relay was dramatically inhibited in cells with defective CV function.

### Involvement of PI(4,5)P2 in Polarity Network and Contractile Vacuole Localization

PI(4,5)P2 has been suggested to be elevated at the cell’s trailing edge in *D. discoideum* and in neutrophils (Janetopoulos et al., 2005; Hind et al., 2016; Iijima et al., 2004). PIs are regulated by different phosphatases and kinases localized at distinct areas in the cell (Balla, 2013; Choi et al., 2016). Phosphoinositide 3-kinases (PI3Ks) are the enzymes that convert PI(4)P and PI(4,5)P2 into PI(3,4)P2 and PI(3,4,5)P3, respectively. PI3Ks localize to the leading edge of migrating cells, while the phosphatase and tensin homolog (PTEN), the phosphatase that utilizes PI(3,4,5)P3 has a substrate, and synthesizes PI(4,5)P2, localizes to the rear of migrating cells (Devreotes and Janetopoulos, 2003; Funamoto et al., 2002; Kriebel et al., 2003; Luo et al., 2003). PTEN activity and the sharp localization at the rear of the polarized cell is, in part, regulated by PI(4,5)P2 binding motif at the N terminus of PTEN (Iijima et al., 2004; Nguyen et al., 2014). Similar morphology has been shown in cells during cytokinesis and in polarized neutrophils (Janetopoulos et al., 2005; Lacalle et al., 2007; Lokuta et al., 2007). The net charge of PI(4,5)P2 is -4, which enables this lipid to contribute to the localization and the activity of various proteins by interacting with their polybasic clusters (Ellenbroek et al., 2011). During the process of aggregation, it is possible that SNARE proteins or other proteins with polybasic motifs, link the CV to the plasma membrane, as discussed below, and contribute to the rearward distribution of the CV to the back of *D. discoideum* cells (Fadil et al., 2022) (Figure 1). Interestingly, this polarized localization can occur in drug treated cells lacking an actin cytoskeleton or functional microtubule network, suggesting that the direct interaction with the PM is responsible for CV localization. CVs were still able to accumulate towards the low side of the chemoattractant gradient, when cells were in the presence of drugs that disrupted the actin cytoskeleton, similar to the movements that can be seen with the reciprocal regulation of PI3K and PTEN (Janetopoulos et al., 2004). The CV and PI(4,5)P2 are elevated in areas of the cell where there are not membrane protrusions (Fadil et al., 2022), which suggests a key role for this lipid in regulating the CV organelle’s localization. The CV disassembles at metaphase, when PI(4,5)P2 levels reach an intermediate level across the entire periphery of the cell, and then begin to reassemble in the furrow during telophase and the initiation of cytokinesis, as PI(4,5)P2 levels elevate (Janetopoulos et al., 2005). The CVs regenerate during the final stages of cytokinesis, with primordial CVs often formed on the trailing edge of the two daughter cells. In budding yeast, the exocyst components, specifically Sec3 and Exo70 (see below) have also been shown to localize to the cleavage furrow throughout cytokinesis (Wu and Guo 2015). Interestingly, in mammalian cells, the exocyst is also enriched at the cleavage furrow and is under the control of Rab11 and RalA (Fielding et al., 2005; Chen et al., 2006; Neto et al., 2013). The amplification of PI(4,5)P2 in the back of the cell during migration and in the cleavage furrow during cytokinesis likely contributes to the CV localization and could provide the proper targeting of proteins needed for regulating exocytosis. This may also assist in the targeted release of smaller vesicles, sometimes termed exosomes, that have also have been postulated to play a role in the release of cAMP at the rear of the cell (Kriebel, et al., 2018). Therefore, in addition to helping position the CV within the cell, elevated levels of PI(4,5)P2 appear to regulate CV fusion with the PM during vegetative growth and at the rear of migrating cells.

### Involvement of PI(4,5)P2 in Contractile Vacuole Exocytosis

PI(4,5)P2, which is the most abundant negatively charged PI on the PM, has multiple roles within a cell, coordinating actin dynamics, contributing to cell polarity and appears to be critical in exocytosis (Di Paolo and De Camilli 2006; Holz et al., 2000; Martin, 2001; Martin, 2015). PI(4,5)P2 interacts with various positively charged proteins in the exocyst complex, suggesting a major role for this PI in regulating the CV organelle’s exocytosis (Essid et al., 2012). This binding of exocyst subunits to the PM delivers the vesicles to the targeted PI(4,5)P2 within the PM for the tethering step. It has been demonstrated that the exocyst subunits Sec3 and Exo70 bind to PI(4,5)P2 at the PM (He et al., 2007; Liu et al., 2007; Zhang et al., 2008; Shewan et al., 2011; Pleskot et al., 2015). Furthermore, it is well-established that polarized exocytosis, which is a multistep vesicular trafficking process, transfers signals and proteins to specific PM sites, and is mediated by PI(4,5)P2 (He and Guo, 2009). SecA, a *D. discoideum* homolog of the yeast Sec1p and the mammalian Munc18 protein, localizes to the CV. Cells with a defect in SecA cannot regulate their osmotic pressure and have a defect in CV discharge (Zanchi et al., 2010). Sec1p and Munc18 proteins are essential for different exocytosis steps as they interact with exocytic SNARE proteins during vesicle docking and fusion, also mediated by PI(4,5)P2 (see below) (Carr et al., 1999; Grote et al., 2000; Shen et al., 2007; Sudhof and Rothman, 2009). SecA is therefore necessary for CV fusion to the PM and water discharge. A recent study suggested that PI(4,5)P2 regulates the trafficking of the CV to the fusion site and cells with a defect in Dd5P4, the enzyme which uses the 5-phosphates of PI(4,5)P2 as substrate, displayed inefficient CV fusion (Luscher et al., 2019).

Exocytosis associated with CV function is regulated by SNARE proteins. There are four homologs of mammalian SNAREs present in *D. discoideum*: vesicle-associated membrane protein 7 (v-SNARE VAMP7), and three t-SNAREs, syntaxin 7, syntaxin 8 and Vti1(Bogdanovic et al., 2000, 2002). It has been reported that VAMP7 and syntaxin 7 are present in the bladder of the CV. The other two SNARE proteins, syntaxin 8 and Vti1 have been seen in both the CV’s bladders and tubular networks (Bennett et al., 2008). Some of the SNARE proteins have also been identified as interactors with PI(4,5)P2 (Murray and Tamm 2009). Clathrin assembly proteins, such as AP180 and AP1, are also related to CV activity. AP180 null cells show unusual large CVs and are osmo-sensitive (Stavrou and O’Halloran, 2006). Moreover, AP180 has been reported to interact with another SNARE, Vamp7B (Wen et al., 2009). AP180 also binds to PI(4,5)P2, through the NH2-terminal homology domain known as ANTH (AP180 N-terminal homology) and assists in clathrin assembly on lipid monolayers (Ford et al., 2001). The ANTH domain is conserved among all members of the AP180 family (Norris et al., 1995; Ye et al., 1995; Hao et al., 1997; Ford et al., 2001; Mao et al., 2001; Stavrou and O’Halloran, 2006). Thus, PI(4,5)P2 has been shown to be a critical regulator of exocytic events in both *D. discoideum* and many eukaryotes, including mammals.

## The General Role of PI(4,5)P2 in Eukaryotic Exocytosis

**FIGURE 2:**
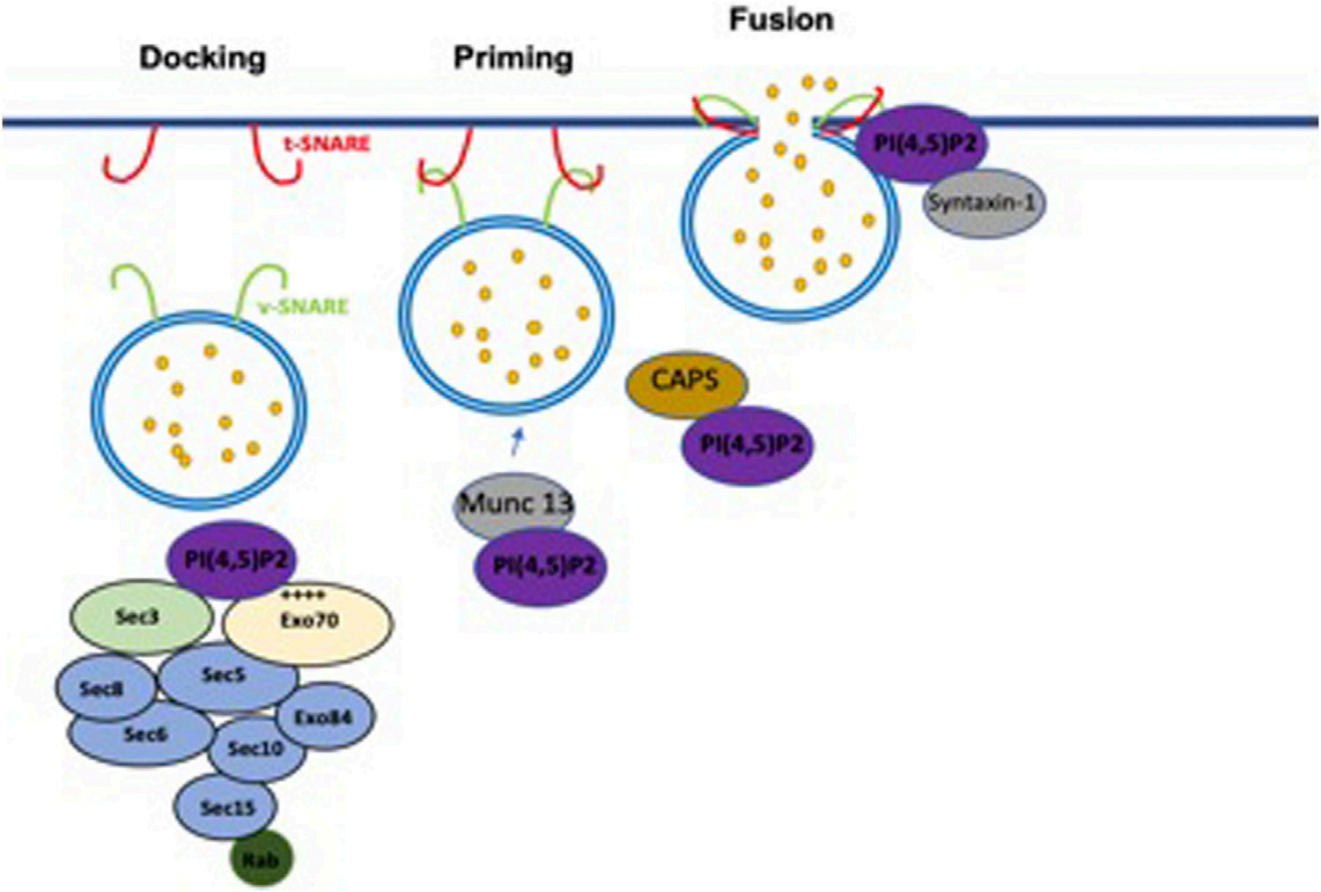
Involvement of PI(4,5)P2 in the exocytosis steps. PI(4,5)P2 is required for mediating tethering of vesicles to the PM. In the priming step, PI(4,5)P2 modulates the vesicles priming by interacting with Munc13 and CAPS. PI(4,5)P2 controls vesicles fusion by interacting with SNARE complex.

In addition to exocytosis being a critical component of CV dynamics, it is also essential for vesicles to eject their contents in metazoans. Exocytosis is a membrane trafficking process that can discharge soluble and insoluble protein contents including neurotransmitters, hormones, and cytokines, as well as many other small molecules and metabolites to the extracellular space. In eukaryotes, this process also mediates the polarized delivery of vesicular trafficking proteins and lipids to specific PM domains. As has been mentioned, several studies show that PI(4,5)P2 and its effectors play critical roles in all steps of exocytosis (Figure 2) (Cremona et al., 1999; Paolo et al., 2004; Holz et al., 2000; James et al., 2008; Milosevic et al., 2005; Martin, 2001; Wenk et al., 2001). To perform this function, secretory vesicles undergo three defined trafficking steps during exocytosis (Figure 2): the docking process for recruiting vesicles to the PM, the priming process for maturing the vesicles, the fusion process to fuse with the PM, and the release of vesicle contents (Voets, 2000; Rettig and Neher, 2002; Sudhof, 2013; Imig et al., 2014). PI(4,5)P2 is capable of engaging in a multitude of cellular functions that are temporally and spatially controlled by the localized distribution of PI(4,5)P2 along the PM (Bai et al., 2004). PI(4,5)P2 has been shown to be enriched in domains at the PM and at the vesicle exocytosis sites (Trexler et al., 2016). Furthermore, the recruitment of enzymes that hydrolyze PI(4,5)P2 at the docking sites results in inefficient docking of the vesicles to the PM (Cremona et al., 1999; Honigmann et al., 2013). This initial priming step of exocytosis is controlled by different proteins and their effectors such as the Ca^2+^-dependent activator protein for secretion (CAPS) and Munc13 (Shin et al., 2010; Kabachinski et al., 2014; Martin, 2015). These proteins are also regulated by PI(4,5P)2, with the PH domain of CAPS binding to PI(4,5)P2 for activation (Grishanin et al., 2004; Kabachinski et al., 2014; Martin, 2015). SNAREs also interact with PI(4,5)P2 for vesicle fusion (Martin, 2001; James et al., 2008).

### Involvement of PI(4,5)P2 With the Exocyst Complex

The exocyst complex has been involved in various cellular processes including exocytosis, cell growth, cytokinesis, cell migration, primary ciliogenesis and tumorigenesis. Furthermore, the exocyst protein complex has a crucial role in polarized membrane protein trafficking (He and Guo, 2009). The exocyst complex (Figure 2) consists of eight subunits (Sec3, Sec5, Sec6, Sec8, Sec10, Sec15, Exo70, and Exo84) that mediate the vesicles’ tethering to the PM and is conserved among the eukaryotic kingdom (He and Guo, 2009; Yeaman et al., 2001). Exocyst subunits interact with each other in pairs: such as Sec3–Sec5, Sec6–Sec8, and Sec10–Sec15 (Guo et al., 2000; Katoh et al., 2015; Heider et al., 2016; Matern et al., 2001; Munson and Novick 2006; Vega and Hsu 2001). The exocyst complex mediates localization and tethering of vesicles to targeted membranes and enables the assembly of SNARE complexes before the fusion step (Pfeffer, 1999; Guo et al., 2000; Waters and Hughson., 2000; Whyte and Munro, 2002). Exo70 is localized near cell-cell contacts on the PM, meaning that Exo70 can mediate PM interaction in these cells independently of the remaining exocyst components (Matern et al., 2001). Indeed, the exocyst needs to interact with the target membrane to achieve tethering function after the delivery of the vesicles. This process appears to be mediated by direct binding of Sec3 and Exo70 subunits with PI(4,5)P2 enriched at the inner leaflet of the PM (He et al., 2007; Liu et al., 2007; Zhang et al., 2008; Shewan et al., 2011; Pleskot et al., 2015). Exo70 exhibits a positively charged surface domain at its C-terminus in mammalian cells which mediates the binding to PI(4,5)P2. Interestingly, the C-terminal sequence of Exo70 is the most evolutionarily conserved region of the protein, suggesting this PI(4,5)P2 is critical for many organisms. The same study has also revealed that Exo70 can recruit the other exocyst components to the PM (Liu et al., 2007). Furthermore, studies on yeast have shown that Exo70 is an effecter of the GTPase Rho3, which plays regulatory roles in actin organization and exocytosis (Adamo et al., 1999; Robinson et al., 1999). The disruption of the Exo70–lipid interaction resulted in exocyst mis-localization and ablation of the enzyme secretion responsible for yeast cell growth. On the other hand, disruption of Exo70–Rho3 interaction did not show any noticeable defects (He et al., 2007). The binding affinity of Exo70 for PI(4,5)P2 is higher than PI(3,5)P2, PI(3)P or PI(4)P (He et al., 2007). The other component that mediates the delivery of the vesicles to the targeted membrane is Sec3. The association between the amino-terminal PH domain of Sec3 and PI(4,5)P2 mediates Sec3’s localization to the PM (Luo et al., 2014). It has been reported that the PH domain of Sec3 also interacts with small GTPases such as Cdc42 and Rho1 (Guo et al., 2000; Zhang et al., 2001; Zhang et al., 2008). Small GTPases and PI(4,5)P2 can synergistically influence the exocyst complex’s position and function at the PM. When both Exo70 and Sec3 are impaired, it is no longer possible to anchor the exocyst complex to the targeted membrane (He et al., 2007). Thus, Exo70 and Sec3 bind to PI(4,5)P2 and function in concert to mediate the association of the exocyst complex with the PM.

### PI(4,5)P2 is the Binding Site for CAPS and Munc-13

CAPS and Munc-13 are major contributors to the priming step in exocytosis of synaptic vesicles and dense-core vesicles (Figure 2) (Wojcik and Brose, 2007; Stevens and Rettig, 2009; James and Martin, 2013). These synaptic vesicles and dense core vesicles play an essential role in neuronal communication and brain development (Rettig and Neher, 2002; Sudhof and Rizo, 2011). It has been reported that Munc-13 is critical for synaptic vesicle exocytosis, while CAPS plays a central role in dense-core vesicles (Augustin et al., 1999; Varoqueaux et al., 2002; Grishanin et al., 2004; Speese et al., 2007; Liu et al., 2008). Moreover, CAPS and Munc-13 have been shown to regulate SNARE assembly with the vesicles and are crucial for exocytosis (Basu et al., 2005; James et al., 2009; Daily et al., 2010; Khodthong et al., 2011; Ma et al., 2011; Wang et al., 2017). CAPS and Munc13 proteins have interconnected C-terminal SNARE protein–binding domains (Koch et al., 2000; Guan et al., 2008; Pei et al., 2009). Deleterious mutations in the PH domain of CAPS result in controlled exocytosis failure (Grishanin et al., 2002). Also, CAPS interacts with syntaxin-1 near its PI(4,5)P2 binding site, indicating that PI(4,5)P2 is an essential co-factor for activating CAPS via binding to its PH domain. On the other hand, Munc13 binds to PI(4,5)P2 in a Ca^2+^-dependent manner through its C2B domain (James et al., 2010; Shin et al., 2010). Moreover, a study on neuroendocrine cells has shown that Munc-13 is cytoplasmic and translocates to PI(4,5)P2-rich PM domains in response to Ca^2+^ influx (Kabachinski et al., 2014; Martin, 2015). In line with this, Munc13-1-GFP translocation to microdomains was blocked by overexpression of the high-affinity PI(4,5)P2-binding PH domain of PLCδ1, demonstrating their affinity for the same PM site (Kabachinski et al., 2014). Indeed, both CAPS and Munc13 can promote the recruitment of vesicles to PI(4,5)P2-rich membranes in a Ca2+-dependent manner (Junge et al., 2004; Zikich et al., 2008; Kabachinski et al., 2016; Kreutzberger et al., 2017).

### PI(4,5)P2 interacts With SNARE Complex

SNARE proteins are the central components of the fusion step, the final process in exocytosis vesicle trafficking (Figure 2). All SNARE family members have a distinctive preserved homolog stretch of 60–70 amino acids, known as the SNARE motif (Barg et al., 2010). Among a large number of SNARE proteins, three complexes were carefully studied and identified. These complexes are Syntaxin-1, synaptosome-associated protein (SNAP-25), and synaptobrevin2/vesicle-associated membrane protein 2 (VAMP2) (Barg et al., 2010; Rickman et al., 2010; Bar-On et al., 2012; Ji et al., 2015). It has been reported that PI(4,5)P2 activates syntaxin-1 promoting assembly with SNAP-25 (Murray and Tamm 2009). Furthermore, syntaxin interactions with PI(4,5)P2 play a positive role in vesicle fusion with the membrane by localizing the protein on the membrane or promoting SNAP-25 interactions (Van den Bogaart et al., 2011). Fusion-competent vesicles in PC12 have been suggested to localize preferentially to PM sites that contain either PI(4,5)P2 domains or PI(4,5)P2 domains co-localized with syntaxin-1 clusters (Van den Bogaart et al., 2011). Other studies demonstrated that a subset of docked vesicles are actually present in PI(4,5)P2-enriched areas where exocytosis occurs under optimal Ca^2+^ influx conditions (Ji et al., 2015; Ji and Lou, 2016). Syntaxin-1 interacts with PIs through a membrane-proximal sequence of basic residues which includes K^260^ARRKK^265^. Importantly, syntaxin-1 clusters were eliminated by treatment of PC12 cells with the 5-phosphatase synaptojanin-1. Synaptotagmin1, another calcium sensor for exocytosis, is anchored to the membrane of secretory organelles which is mediated by PI(4,5)P2 clusters in plasma domains (Aoyagi et al., 2005; Gandasi and Barg, 2014; Murray and Tamm, 2009). Moreover, the interaction between PI(4,5)P2 and Synaptotagmin increases the excitation-secretion in response to Ca^2+^ (Bai et al., 2004). SNARE proteins therefore play a critical role in the fusion step and are regulated by local PI(4,5)P2 levels as they modulate vesicle exocytosis.

## Discussion

The contractile vacuole is part of the polarity circuit and is enriched at the rear of migrating cells, with this localization being critical for cAMP secretion. PI(4,5)P2 is the most abundant PIs in the PM and has been shown to be elevated at the rear of the cell and in areas reciprocally regulated with PI(3,4,5)P3 and membrane protrusions. PI(4,5)P2 has multiple roles in the cell, and is intimately involved in membrane trafficking, endocytosis and exocytosis. The regulatory factors governing exocytosis are highly conserved across species and as highlighted in this review are relevant to many mammalian cell trafficking pathways. The rear CV enrichment and exocytosis described here may be related to this migracytosis mechanism recently described which has been implicated in cell-cell communication in mammalian cells. Migrasomes exist in many cell types such as normal rat kidney cells, macrophages, primary neurons, human breast cancer cells, and embryonic stem cells (Ma et al., 2015). Migracytosis is a cell migration-dependent mechanism for releasing cellular contents by migrasome organelles which localize at the rear of the cell and play a potential role in cell-cell communication.

PI(4,5)P2 pathway dysregulation and failure of proper exocytosis has been identified in many different diseases such as Lowe syndrome, neuronal disorders and various forms of cancer (Dannemann et al., 2012; Kim et al., 2017; Prosseda et al., 2017; Raghu et al., 2019). Lowe oculocerebrorenal syndrome, a congenital disease characterized by low IQ, and defective kidney proximal tubule resorption, is caused by a defect in the OCRL gene. OCRL is an inositol polyphosphate 5-phosphatase that hydrolyzes the 5-phosphate of PI(4,5)P2 into PI4P (Prosseda et al., 2017). Interestingly, a recent study in *D. discoideum* has shown that OCRL-like protein of *D. discoideum*, Dd5P4, is recruited to the CV membrane upon the kiss-and-run exocytic event (Luscher et al., 2019). Therefore, it is possible that the exocytic function of ORCL contributes to the pathological process of Lowe syndrome. Chediak-Higashi syndrome (CHS) is an autosomal human disorder characterized by immunodeficiency and the formation of giant lysosomes or lysosome-related organelles. In *D. discoideum,* the homolog to one of the Chediak-Higashi syndrome (CHS) proteins is LvsA, which interestingly enough labels the CV bladder and remains associated throughout the discharge phase until fusion with the PM (Gerald et al., 2002). In addition, it also seems plausible that the localized fusion that occurs with CV discharge is correlated with the small extracellular vesicles documented during *D. discoideum* streaming (Kreibel, et al., 2018). Extracellular vesicles are critical for many aspects in tumor progression (Xavier et al., 2020), so studying these processes is of utmost importance. Given that PI(4,5)P2 levels are critical to so many processes in the cell, there are certain to many critical roles and potential therapeutic interventions that can be performed by regulating PI(4,5)P2 levels. Understanding the basic function of PI(4,5)P2 in CV ejection or neurotransmitter release may even help with diseases of the brain. PI(4,5)P2 levels are decreased in the brain of Alzheimer’s patients, for instance, although the exact role in Alzheimer’s disease has yet to be elucidated (Stokes et al., 1978; Arancio., 2008). There are many steps in CV localization and function where PI(4,5)P2 seems to be required, so understanding the basic functions to their targeting and function should enhance our understating of exocytosis-related diseases.
